# Fractures and skin lesions in pediatric abusive head trauma: a forensic multi-center study

**DOI:** 10.1007/s00414-021-02751-4

**Published:** 2021-12-03

**Authors:** Katharina Feld, Tim Ricken, Dustin Feld, Janine Helmus, Maria Hahnemann, Sebastian Schenkl, Holger Muggenthaler, Heidi Pfeiffer, Sibylle Banaschak, Bernd Karger, Daniel Wittschieber

**Affiliations:** 1 Institute of Legal Medicine, University Hospital Cologne, Cologne, Germany; 2 Institute of Legal Medicine, University Hospital Münster, Münster, Germany; 3 adiutaByte GmbH, Business Campus, Sankt Augustin, Germany; 4 Institute of Legal Medicine, University Hospital Essen, Essen, Germany; 5 Division of Radiology, Medical Practice, Jena, Germany; 6 Institute of Legal Medicine, Jena University Hospital, Friedrich Schiller University, Am Klinikum 1, 07747 Jena, Germany

**Keywords:** Child abuse, Skeletal survey, Hematoma, Fracture, Injury pattern

## Abstract

Abusive head trauma (AHT) and its most common variant, the shaken baby syndrome (SBS), are predominantly characterized by central nervous system-associated lesions. Relatively little data are available on the value of skeletal and skin injuries for the diagnosis of SBS or AHT. Thus, the present study retrospectively investigated 72 cases of living children diagnosed with the explicit diagnosis of SBS during medico-legal examinations at three German university institutes of legal medicine. The risk of circular reasoning was reduced by the presence of 15 cases with confession by perpetrators. Accordingly, the comparison with the 57 non-confession cases yielded no significant differences. Skeletal survey by conventional projection radiography, often incomplete, was found to be performed in 78% of the cases only. Fractures were found in 32% of the cases. The skull (43%) and ribs (48%) were affected most frequently; only 8% of the cases showed classic metaphyseal lesions. In 48% of the cases, healing fractures were present. Skin lesions (hematomas and abrasions) were found in 53% of the cases with the face (76%), scalp (26%), and trunk (50%) being the major sites. In 48% of the cases, healing skin lesions were observed. Nearly 80% of the cases with fractures also showed skin lesions. The data prove that SBS is frequently accompanied by other forms of physical abuse. Therefore, skeletal survey is indispensable and should always be done completely and according to existing imaging guidelines if child abuse is suspected.

## Introduction

Head trauma is a crucial factor for mortality in both adults [1–3] and young children [4–6]. The pediatric abusive head trauma (AHT), which is mainly described in infants and toddlers up to the age of 2 years, has an incidence of 20 to 40 per 100,000 live births younger than 1 year [7–13]. Long-term damages can be found in up to 96% of cases, and the lethality rate ranges between 13 and 36% [5]. Throughout the entire recent body of literature on that topic, AHT is generally understood as an umbrella term comprising different types of violence against a child’s head such as violent shaking of the child (referred to as shaken baby syndrome [SBS]), mere blunt force or impact trauma, or combinations of both (sometimes also referred to as shaken impact syndrome [SIS]).

SBS (and AHT in general) is predominantly related with central nervous system-associated lesions such as injuries of the brain parenchyma, retinal hemorrhages, subdural fluid collections (such as hematomas or hygromas), bridging vein thromboses, or spinal injury [5, 6, 14–21]. Besides, several non-neurologic findings, especially fractures and skin lesions, may be encountered and are considered results of both AHT and other forms of physical abuse independent of AHT [22–27]. However, detailed and comparable data on fractures and skin lesions in cases of AHT are relatively sparse. Another problem in AHT studies relates to circular reasoning: If the inclusion criterion for the diagnosis of AHT is solely based on generally accepted medical findings (e.g., subdural hematoma), it is likely that these findings are found to be characteristic features of AHT.

Therefore, the present study aims at investigating the presence of fractures and skin lesions and their value for the diagnosis of SBS and AHT, respectively. To this issue, a study cohort of 72 SBS cases was compiled using a multi-center approach and a retrospective 10-year period. To reduce the danger of circular reasoning, cases with and without confession by perpetrators were compared. Concordant results independent of a confession would strongly support a correct diagnosis of SBS/AHT also in the non-confession cases.

## Material and methods

### Compliance with ethical standards

The present study has been approved by the ethical boards of all participating institutions, which are the university hospitals of the North Rhine-Westphalian cities of Cologne, Essen, and Münster, reference number: 2014–658-f-N (ethics committee of the Medical Association of Westfalen-Lippe and the Westphalian Wilhelms University). In addition, the study has been approved and supported by the Ministry of Justice of the Federal State of North Rhine-Westphalia and the Attorney General's Office (file number: 1410 E -II. 75/17). Experiments on humans or animals were not performed for this study.

### Case collection and data acquisition

All expert opinions on living children written at the university institutes of legal medicine of the German cities of Cologne, Essen, and Münster between 2006 and 2015 by order of courts or public prosecutors were retrospectively reviewed with respect to the major diagnosis of “Schütteltrauma” (which is the German equivalent to “shaken baby syndrome,” SBS), the most frequent diagnosis in the context with AHT in Germany. In these cases, violent shaking can be expected to represent the priority cause of the head trauma but not necessarily the only one, i.e., additional blunt force trauma to the head could have occurred as well, according to the diagnosis that can be referred to as SIS.

This approach was chosen due to several reasons: First, medico-legal expert opinions represent the best reference standard for the diagnosis and forensic assessment of AHT cases in Germany. Second, the fact that the children were all alive at the time of the forensic examination warranted the presence of comprehensive diagnostic data from the treating hospital (radiology, ophthalmology, laboratory medicine, etc.) which is frequently not the case in children primarily occurring as forensic autopsy cases with sudden unexpected death that turned out to be a death from AHT. Third, as AHT is predominantly diagnosed in living children at hospital (and not in the autopsy room), we aimed on the comparability of our data with cases from the clinical routine, i.e., AHT cases that clinicians and clinical forensic physicians (or child abuse pediatricians) are typically confronted with.

The complex diagnosis of SBS and AHT, respectively, in medico-legal expert opinions of the participating institutes is based on both the consensus of a multi-headed clinical team after a comprehensive diagnostic process within the treating hospital and the additional evaluation by an independent university institute of legal medicine. The diagnostic process comprises a number of standardized clinical, radiological, laboratory, and, if necessary, other investigations and tests in order to detect findings confirming the suspected diagnosis and— vice versa—to rule out the relevant differential diagnoses such as metabolic, hematologic, neoplastic, or infectious diseases, or birth trauma. Confessions by perpetrators were not known to any medical personnel, neither during the hospitalization nor while preparing the medico-legal expert opinions; thus, such data were not able to bias the diagnostic process.

With respect to SBS, being a very common variant of AHT, the following findings were considered typical, but neither obligatory nor evidentiary: acute encephalopathy (as revealed by typical neurological symptomatology and/or primary traumatic and/or secondary hypoxic-ischemic damage of the brain parenchyma in neuroimaging), subdural collections (with or without other additional extra-axial findings in neuroimaging), retinal hemorrhages, spinal trauma, missing or inadequate trauma anamnesis.

During hospitalization, the diagnostic process is closely accompanied by at least one experienced forensic physician of an independent university institute of legal medicine. The forensic physician always performed an independent medico-legal physical examination of the living child, and, if necessary, advised the clinical team with his/her special expertise for diagnostics of child abuse. In sum, the complete clinical records, the histories reported by caregivers during police interrogations, and other initial criminal investigation results were considered for the final expert opinion.

In total, 72 cases were identified for further analysis.

### Analysis of the data

Two specialists with board certification in legal medicine with experience in assessing SBS/AHT cases (KF and DW) consensually analyzed the medico-legal expert opinions as well as the available clinical records regarding all demographic and forensically relevant data. Subsequently, fractures and skin lesions were recorded and analyzed in more detail. The fractures were determined by means of the written radiological reports of the radiographic, computed tomography, and magnetic resonance imaging studies. The skin lesions were recorded by means of the detailed descriptions of the entire skin surface, which are part of each medico-legal expert opinion and also contain negative findings (i.e., skin regions without visible injuries).

Based on a previous analysis of the associated criminal proceedings [28], the total study cohort was subsequently subdivided into “non-confession cases” (cases without confessed perpetrators) and “confession cases” (cases with confessed perpetrators). Thereby, homogeneity of the whole study group can be demonstrated independent of a confession, which strongly supports a correct diagnosis also in the non-confession cases and thus reducing circular reasoning.

### Statistical analysis

The programming language and free software environment for statistical computing “R” (version 3.6.1) was used for statistical evaluations, such as basic statistical functions like mean(x, …), median(x, na.rm = FALSE, …). *p* values were estimated by the Binomial {stats} functions, e.g., dbinom(x, size, prob, log = FALSE). *p* values < 0.05 were considered statistically significant.

## Results

Table 1 shows the general characteristics of the study cohort. There were no significant differences between the groups without and with confession (all *p* values > 0.05). Instead, all sub-groups showed nearly exactly the same proportions as to the parameters considered (e.g., male–female ratio, mean and median age, or occurrence of fractures and skin lesions), thereby demonstrating homogeneity of the study group independent of a confession.

**Table 1:** General characteristics of the study cohort

	Total study cohort	Non-confession cases	Confession cases	*p *value^3^
*n*	72	57	15	0.55
Sex, *n* (%)				
Male	48 (67)	38 (67)	10 (67)	0.56
Female	24 (33)	19 (33)	5 (33)	0.58
Age in months (mean; median; range)				
Mean	4.4	4.4	4.4	-
Median	3	3	3	-
Range	0–30	0–30	1–5	-
Mode of delivery, *n* (%)				
Unknown	30 (42)	25 (44)	5 (33)	0.38
Vaginal birth	26 (36)	18 (32)	8 (53)	0.16
Cesarean section	14 (19)	12 (21)	2 (13)	0.42
Forceps birth	2 (3)	2 (3)	0	0.63
Diagnostic findings, n (%)^1^				
Subdural fluid collections^2^	71 (99)	56 (98)	15 (100)	0.52
Epidural hematoma	2 (3)	2 (3)	0 (0)	0.63
Retinal hemorrhages	57 (79)	44 (77)	13 (87)	0.41
Unilateral	9/57 (16)	7 (12)	2 (13)	0.59
Bilateral	48/57 (84)	37 (65)	11 (73)	0.42
Fracture(s)	23 (32)	18 (32)	5 (33)	0.54
Skin lesion(s)	38 (53)	30 (53)	8 (53)	0.55

*n* number of cases, % percent

^1^ Numbers do not add up to *n*=72 (100%) due to multiple diagnostic imaging procedures and diagnostic findings

^2^ Comprising subdural hematomas, subdural hygromas, and/or subdural hematohygromas

^3^ Comparison between non-confession and confession cases

**Fig. 1:**
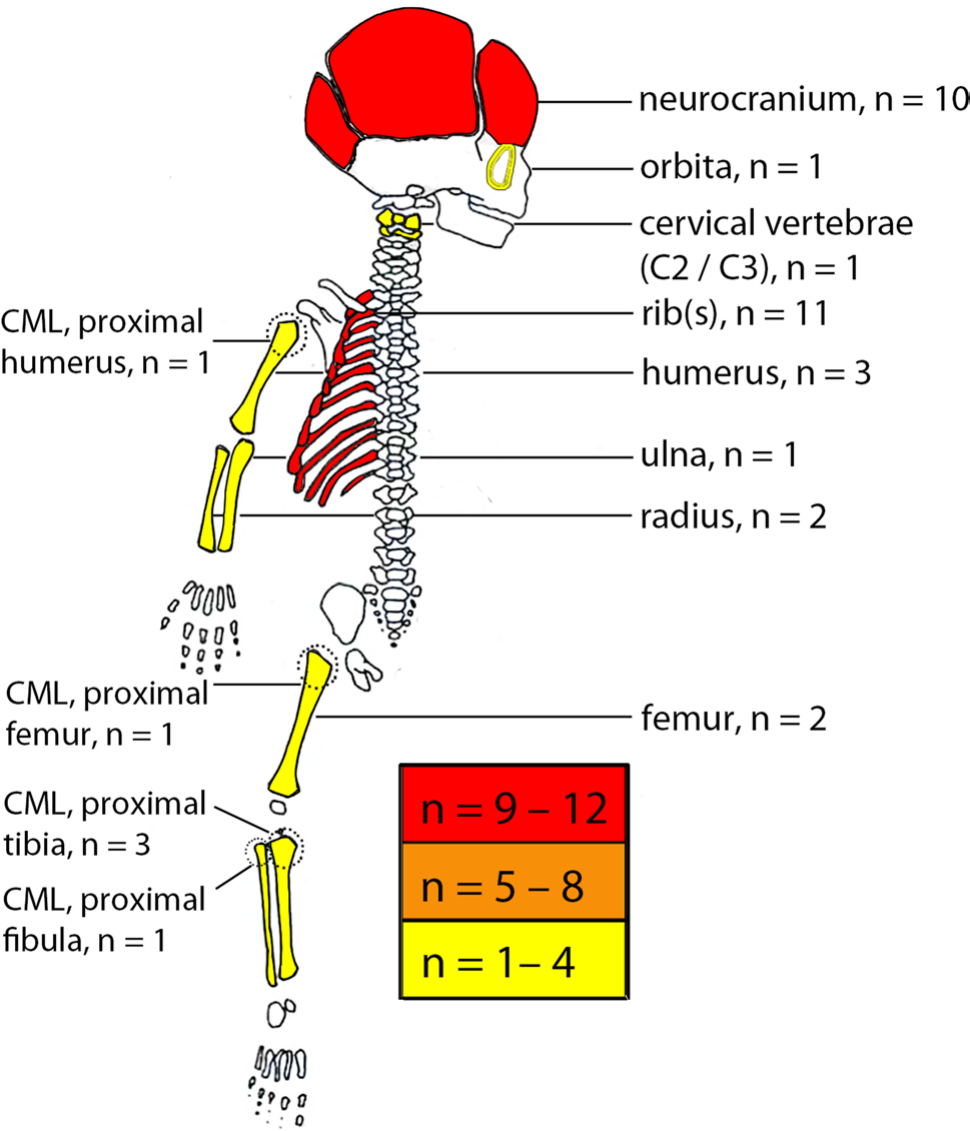
Synopsis of the frequency and locations of fractures found in 23 AHT cases. Paired locations are shown only once, but apply for both body sides. *n*=number of cases

Fractures were found in 23 out of 72 AHT cases (32%). The detailed analysis of the fractures is summarized in Table 2. The fracture characteristics did not show any significant differences between non-confession and confession groups (*p* values > 0.05), except for one aspect (multiple fractures with two different ages). The diagnostic imaging procedures performed on the children of the study cohort are presented in Table 3. The general frequency of the fracture locations is graphically visualized in Fig. 1. The osseous pelvis was the only skeletal region without fractures.

**Table 2:** Characteristics of the fractures

	Total study cohort (*n* = 72)	Non-confession cases (*n* = 57)	Confession cases (*n* = 15)	*p* value^2^
Number of cases with fractures, *n* (%)	23/72 (32)	18/57 (32)	5/15 (33)	-
Fractured bones, *n* (%)				
Single fracture	12/23 (52)	11/18 (61)	1/5 (20)	0.23
—Single rib	3/12 (25)	3/11 (28)	-	0.48
—Temporal bone	2/12 (17)	2/11 (18)	-	0.61
—Parietooccipital region	2/12 (17)	2/11 (18)	-	0.61
—Femur	2/12 (17)	1/11 (9)	1/1 (100)	0.39
—Frontal bone	1/12 (8)	1/11 (9)	-	0.78
—Orbital	1/12 (8)	1/11 (9)	-	0.78
—Spine	1/12 (8)	1/11 (9)	-	0.78
Multiple fractures	11/23 (48)	7/18 (39)	4/5 (80)	0.20
—Multiple ribs	4/11 (37)	3/7 (44)	1/4 (25)	0.62
—Multiple ribs + parietal bone	1/11 (9)	1/7 (14)	-	0.78
—Multiple ribs + both parietal bones + occipital bone + radius + ulna	1/11 (9)	1/7 (14)	-	0.78
—Multiple ribs + humerus	1/11 (9)	-	1/4 (25)	0.22
—Multiple ribs + humerus + femur	1/11 (9)	-	1/4 (25)	0.22
—Tibia + fibula	1/11 (9)	1/7 (14)	-	0.78
—Proximal tibia + distal tibia + humerus	1/11 (9)	1/7 (14)	-	0.78
—Humerus + radius + tibia	1/11 (9)	-	1/4 (25)	0.22
Classical metaphyseal lesions, *n* (%)	6/72 (8)	4/57 (7)	2/15 (13)	0.39
Proximal tibia	3/6 (50)	2/4 (50)	1/2 (50)	0.52
Proximal fibula	1/6 (17)	1/4 (25)	-	0.78
Proximal humerus	1/6 (17)	-	1/2 (50)	0.22
Proximal femur	1/6 (17)	1/4 (25)	-	0.78
Age of fractures, *n* (%)				
Healing fracture(s)^1^	11/23 (48)	7/18 (28)	4/5(80)	0.20
Multiple fractures with the same age	6/23 (26)	5/18 (28)	1/5 (20)	0.61
Multiple fractures with two different ages	4/23 (17)	1/18 (6)	3/5 (60)	0.03*
Multiple fractures with three different ages	1/23 (5)	-	1/5 (20)	0.22

*n* number of cases, % percent

^1^ Cases with a single fracture (*n* = 3) or cases with multiple fractures (*n* = 8) that appeared older than the day of the X-ray examination

^2^ Comparison between non-confession and confession cases

^*^ *p* < 0.05

**Table 3:** Diagnostic imaging procedures

	Total study cohort (*n* = 72)	Non-confession cases (*n* = 57)	Confession cases (*n* = 15)
Neuroimaging, *n* (%)^1^	72/72 (100)	57/57 (100)	15/15 (100)
Cranial computed tomography	39/72 (54)	31/57 (54)	8/15 (53)
Cranial magnetic resonance imaging	66/72 (92)	51/57 (89)	15/15 (100)
Cranial ultrasound	55/72 (76)	41/57 (72)	14/15 (93)
Conventional projection radiography, *n* (%)^1^	57/72 (79)	42/57 (74)	15/15 (100)
Full body (“babygram”)^2^	11/72 (15)	10/57 (18)	1/15 (7)
Skull	19/72 (26)	18/57 (32)	1/15 (7)
Arms	36/72 (50)	24/57 (42)	12 /15 (80)
Legs	36/72 (50)	27/57 (47)	9/15 (60)
Thorax	44/72 (61)	30/57 (53)	14/15 (93)
Spine	27/72 (38)	19/57 (33)	8/15 (53)
Pelvis	21/72 (29)	16/57 (28)	5/15 (33)
Other	5/72 (7)	3/57 (5)	2/15 (13)

*n* number of cases, % percent

^1^ Numbers do not add up to *n* = 72 (100%) due to multiple procedures

^2^ Obsolete

**Fig. 2:**
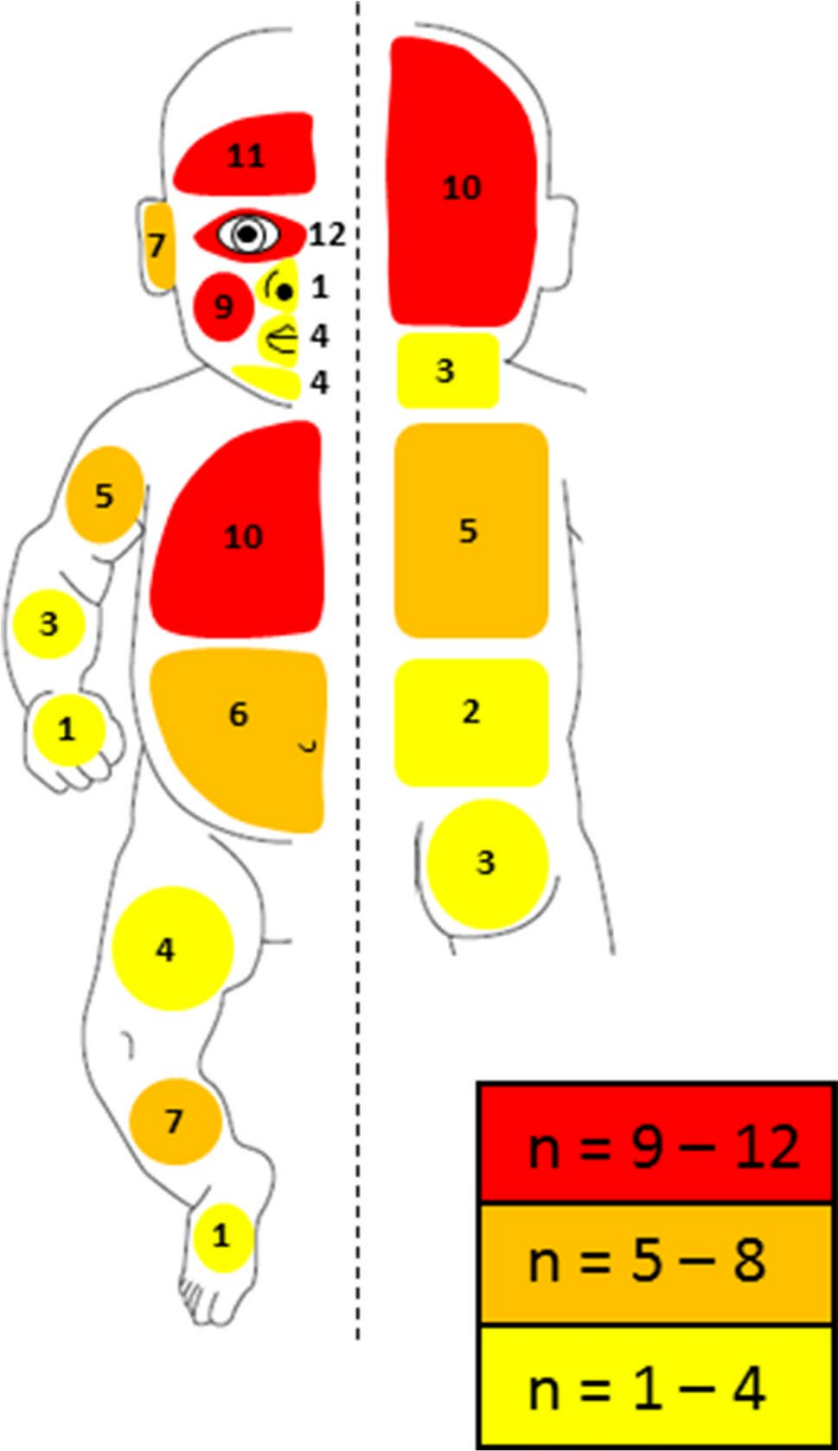
Graphical visualization of the frequency of skin lesions. The numbers at the different body regions (front side, left; back side, right) indicate case numbers of the total study cohort. The case numbers shown at the extremities include both front and back sides

Skin lesions were ascertained in 38 out of 72 AHT cases (53%). Table 4 and Fig. 2 show the characteristics of the skin lesions. Most skin regions did not reveal any significant differences between non-confession and confession groups (*p* values > 0.05). However, in 4 skin regions (the ears, chin, buttocks, and thigh) significant differences were found between the 2 groups. Genitals and feet represented the only regions without skin lesions.

**Table 4:** Characteristics of the skin lesions

	Total study cohort (*n* = 72)	Non-confession cases (*n* = 57)	Confession cases (*n* = 15)	*p*-value^3^
Number of cases with skin lesions, *n* (%)	38/72 (53)	30/57 (53)	8/15 (53)	0.56
Single lesion	25/38 (66)	14/30 (47)	3/8 (38)	0.51
Multiple lesions	13/38 (34)	16/30 (53)	5/8 (62)	0.46
Locations of the skin lesions, *n* (%)^1^				
Face	29/38 (76)	23/30 (77)	6/8 (75)	0.59
—Forehead	11/29 (38)	9/23 (39)	2/6 (33)	0.58
—Eye region(s)	12/29 (41)	7/23 (30)	5/6 (83)	0.09
—Cheeks	9/29 (31)	7/23 (30)	2/6 (33)	0.59
—Nose	1/29 (3)	-	1/6 (17)	0.21
—Ears	7/29 (24)	3/23 (13)	4/6 (67)	0.04*
—Mouth	4/29 (14)	3/23 (13)	1/6 (17)	0.61
—Chin	4/29 (14)	1/23 (4)	3/6 (50)	0.03*
Scalp	10/38 (26)	10/30 (33)	-	0.09
Neck	3/38 (8)	2/30 (7)	1/8 (13)	0.51
Trunk	19/38 (50)	14/30 (47)	5/8 (63)	0.37
—Chest, front and back side	4/22 (18)	2/16 (13)	2/6 (33)	0.20
—Chest, front side only	6/22 (27)	5/16 (31)	1/6 (17)	0.63
—Chest, back side only	1/22 (5)	1/16 (6)	-	0.79
—Abdomen	6/22 (27)	5/16 (31)	1/5 (20)	0.63
—Lower back	2/22 (9)	1/16 (6)	1/5 (20)	0.38
Genitals	-	-	-	-
Buttocks	3/38 (8)	-	3/8 (38)	0.01*
Upper arm	5/38 (13)	4/30 (13)	1/8 (13)	0.72
Lower arm	3/38 (8)	1/30 (3)	2/8 (25)	0.11
Hands	1/38 (3)	1/30 (3)	-	0.79
Thigh	4/38 (11)	1/30 (3)	3/8 (38)	0.03*
Knee	1/38 (3)	1/30 (3)	-	0.79
Lower leg	7/38 (18)	5/30 (17)	2/8 (25)	0.45
Feet	-	-	-	-
Types of skin lesions, *n* (%)^1^				
Subcutaneous hematoma	25/38 (66)	20/30 (67)	5/8 (62)	0.56
Intracutaneous hematoma	9/38 (24)	6/30 (20)	3/8 (38)	0.29
Abrasions	8/38 (21)	4/30 (13)	4/8 (50)	0.07
Erythema	8/38 (21)	6/30 (20)	2/8 (25)	0.53
Edema	6/38 (16)	5/30 (17)	1/8 (13)	0.63
Petechiae	5/38 (13)	3/30 (10)	2/8 (25)	0.28
Pustule	1/38 (3)	1/30 (3)	-	0.79
Healing lesions^2^	18/38 (48)	14/30 (47)	4/8 (51)	0.54

*n* number of cases, % percent

^1^ Numbers do not add up to *n* = 72 (100%) due to multiple locations/lesions

^2^ Include non-acute healing of subcutaneous hematomas and/or abrasions

^3^ Comparison between non-confession and confession cases

^*^ *p* < 0.05

The co-existence of skin lesions and fractures was analyzed separately (Table 5).

**Table 5:** Co-existence of fractures and skin lesions

	Total study cohort	Non-confession cases	Confession cases
Cases with fractures	23	18	5
Fractures only/no skin lesions	5	4	1
Cases with skin lesions	38	30	8
Skin lesions only/no fractures	20	16	4
Fractures and skin lesions	18	14	4

The numbers indicate case numbers

## Discussion

### General considerations

Using data of 72 SBS cases from 3 German university institutes of legal medicine of a 10-year period, the present study focused on fractures and skin lesions as to their value for the diagnosis of SBS and AHT, respectively. The strength of the study is that confessions by perpetrators could be used for validation. The confessions of the present study were previously disclosed [28]. Thus, the danger of circular reasoning, which otherwise can be a limitation in AHT studies, could be reduced by comparison of 15 cases with and 57 cases without confession by perpetrators. We are aware that confessions by perpetrators cannot be considered scientific evidence because confessions may be incomplete, trivialized, or even wrong. However, confessions can at least be used as strong additional indicators for AHT and they may provide unique insights into the nature and etiology of AHT.

Of course, “reduction” (of circular reasoning) does not mean “exclusion.” This could probably only be achieved by the presence of one or more testimonies by independent witnesses. However, such data are very rare and did not occur in cases of the present study. Frequently, the perpetrator belongs to the close family environment [28] and is often alone with the child during the act of maltreatment. Furthermore, the young victims are mostly incapable of reporting on incidents themselves. Therefore, as similarly performed by others [29], it was only possible to reduce circular reasoning by using confessions of perpetrators and comparing this group to the non-confession group.

Indeed, we found nearly no significant differences between the confession and non-confession groups regarding general and medical data. The proportions of fractures and skin lesion were even identical in both groups. In terms of the 4 skin regions (the ears, chin, buttocks, and thigh) and 1 fracture aspect (multiple fractures with two different ages), where significant differences were found between the 2 groups, the small case numbers of the sub-groups have to be considered. In general, the number of children included in the study may have been too small to reliably detect further differences between the 2 groups. Nevertheless, the striking similarity in the vast majority of all characteristics of both groups strongly suggests that the causal mechanisms may well have been the same.

### Fractures

Skeletal survey by conventional projection radiography was performed in 78% of the cases only and turned out to be incomplete in many cases. This limitation of the study is due to the retrospective study design and can also be encountered in similar studies investigating fractures in cases of AHT. For instance, Sieswerda-Hoogendoorn et al. stated that in 44% of their cases one or more of the images were missing [24].

Our study revealed the presence of one or multiple fractures in approx. one-third of the cases (32%). This finding is basically in line with previous AHT studies which described one or more fractures in 9–58% of the cases, depending on inclusion criteria:

- 9% post-mortem and 24% ante-mortem in Hughs-Roberts et al. (2012) [22],
- 17% in Kemp et al. (2003) [30],
- 22% in non-confession cases and 37% in confession cases in Adamsbaum et al. (2010) [29],
- 25% in Vinchon et al. (2005) [31],
- 41% in Sieswerda-Hoogendorn et al. (2014) [24],
- 46% in age < 2 years in Nuño et al. (2019) [32],
- 50% in Kleinman et al. (2011) [23],
- 52% in Hettler and Greenes (2003) [33],
- 56% in Bechtel et al. (2004) [34], and
- 58% in Ettaro et al. (2004) [35].

When comparing these data, it has to be considered that the main focus of our study cohort is on SBS cases (i.e., AHT predominantly caused by violent shaking of the child). Therefore, missing AHT cases with mere blunt force trauma against a child’s head, that may inherently rather be accompanied by skull fractures, may have most probably led to an underrepresentation of skull fractures in our study.

Likewise in accordance with previous studies, fractures of the skull and ribs (single or serial) accounted for the most frequent bony lesions in our study cohort (*n* = 10/23, 43%; and *n* = 11/23, 48%). Fractures of these anatomical structures are well-known to be associated with AHT and are strong predictors of AHT [36–39]. Compression of the thorax during violent shaking of the firmly held child may explain the occurrence of rib fractures in our cases, whereas the generation of skull fractures requires an impact trauma of the head—either in conjunction with the violent shaking or independently of it. Despite the rather large number of skull and rib fractures, it is noteworthy for casework in clinical forensic medicine that fractures were *not* found in the majority of our cases (55/72 cases, 76%). Hence, skull and rib fractures may serve as important indicators for AHT, but the lack thereof cannot be used as indication against such a diagnosis (sometimes suggested in court).

In our fracture analysis, special attention was paid to classic metaphyseal lesions (CML) because this special type of transversal fractures is considered highly specific for abuse [40]. CML are predominantly due to torsional and tractional forces caused by twisting, pulling, or compressing the extremities of infants [41]. It has been suggested that the acceleration/deceleration forces occurring when an infant is violently shaken may be sufficient, on their own, to cause CML of the extremities [40]. Our data revealed 6 out of 72 cases (8%) with CML, of which only 1 fracture was located at the proximal humerus. This finding disagrees with recent data by Adamsbaum et al. (2019) [41] who found “CML of the shoulder” in 9 out of 24 AHT cases (37.5%). Their study also demonstrated that, unlike more typical CML locations (particularly at the ankle and knee regions), “CML of the shoulder” were the only CML that was significantly associated with subdural hematomas. The authors discussed that this finding might be related to the violent manipulations of the infant’s shoulders during shaking. Our data may be explained by incomplete X-ray image sets or missing awareness to CML during image analysis. Therefore, it might be promising to focus on CML of the shoulder in forthcoming AHT studies.

A spinal fracture was found in 1 case. This fracture also comprised an anterolisthesis of the 2^nd^ vertebral body onto the 3^rd^ one, which suggests a traumatic effect of violent shaking with corresponding shearing forces to the cervical spine. In this context, it is noteworthy that, unlike spinal subdural hematomas or spinal ligamentous injury, fractures of the spine appear to occur very infrequently in AHT [42].

The other skeletal lesions of our study cohort, such as transverse or diagonal fractures of the diaphyses of the long bones (3 × humerus, 1 × ulna, 2 × radius, 2 × femur), cannot be attributed to the process of violent shaking or AHT in general. Therefore, they can be regarded as consequences of additional and AHT-independent trauma.

Further evidence for the abusive nature of the fractures is provided by the age of the fractures observed. Healing fractures were found in nearly half of all cases with fractures (48%). When multiple fractures were present, 4 cases showed fractures with two different ages, and 1 case even revealed fractures with three different ages suggesting at least 3 different maltreatment events.

### Skin lesions

More than half of the cases (53%) showed either single or multiple skin lesions. The head was affected in more than two-thirds of the cases, which can be expected in a study cohort of pediatric head injuries. Among the different head regions, the face (*n* = 29; especially the forehead, the eye regions and the cheeks) and the scalp (*n* = 10) were found to be the major sites. These locations are usually not covered by clothes. Therefore, intra- and subcutaneous hematomas or abrasions should be detectable comparatively easily. In terms of the scalp, thorough inspection sometimes requires combing dense hair aside in order to detect the skin lesions.

The second most concerned body part was the upper trunk with skin lesions at the front and back sides of the chest in approx. one-third of the cases with skin lesions. Subcutaneous hematomas in these locations can be expected in our study cohort due to the known pathophysiological mechanism of holding the child firmly around its chest during violent shaking. However, similar to skull and rib fractures, skin lesions of the chest were *not* found in the majority of the cases (61/72 cases, 85%). This may be due to the (rather flat) pressure exerted by the comparatively large hands of a perpetrator on the small thorax and the number of textile layers the child wore during violent shaking. Thus, spot-like hematomas on the chest due to pressure by fingers may support the diagnosis of SBS (or AHT in general), but the lack thereof cannot be considered an indication against such a diagnosis (likewise sometimes suggested in court).

Compared to fractures, recent study data on skin lesions in cases of AHT are relatively sparse. Feldman et al. (2015) investigated 383 children with AHT and found extracranially located acute bruises or skin injuries in 54.3% (*n* = 183/337) of the cases, extracranially located healing skin injuries in 20.0% (*n* = 66/330), and scalp soft-tissue swelling in 26.3% (*n* = 88/334) [25]. Amagasa et al. (2018) studied 57 Japanese AHT cases and observed “bruising other than head” in 18% (*n* = 10/57) of the cases, and “scalp findings” in 25% (*n* = 14/57) [43]. In their study on prior opportunities to detect abuse in 232 children with AHT, Letson et al. (2016) described acute bruise in 55% (*n* = 127/232) of the cases, healing bruise in 16% (*n* = 38/232), and scalp soft-tissue injury in 28% (*n* = 65/232) [26]. The authors identified bruising with 11.7% as the 3^rd^ most common prior opportunity after vomiting (31.6%) and prior contact to child protective services (24.0%) [26].

With respect to child physical abuse in general, it is well-known that children who sustain severe physical abuse were found to have had a prior “sentinel bruise” [44–46]. Pierce et al. recommended looking for “red flags” by applying the “TEN-4 FACES P” bruising rule [47, 48]. According to this rule, bruises on the trunk (T), ears (E), and neck (N) in children under 4 years of age as well as any bruise in children under 4 months of age should raise concern for physical abuse. Also bruises on the frenulum (F), auricular area (A), cheek (C), eyes (E), and sclera (S) as well as patterned (P) bruising should raise concerns. Many of these regions— especially the trunk, ears, cheek, and eyes— were predominantly concerned in our study cohort as well. Our data suggest that skin lesions of forehead and scalp may play a role for the early recognition or prevention of AHT cases. In accordance with Sheets et al. [49] and other authors, our data confirm that external injuries (so-called sentinel injuries) are of high value as a predictive factor for more severe maltreatment.

The missing control group of the present study could be considered another limitation. However, due to multiple reasons, this is always quite a challenge in the present context. Possibly, one would strive for creating a control cohort consisting of unambiguously accidental head trauma cases of the same age range with additional presence of subdural hematomas to compare patterns of fractures and skin lesions. However, a major problem was that those cases do frequently not occur as living cases at forensic departments in Germany, i.e., in the context of clinical forensic medicine, which is the basis of the SBS/AHT cohort of the present study, as explained in the “Material and methods” section in more detail.

### Coexistence of fractures and skin lesions

Only 5 out of 23 cases with fractures (22%) did not reveal any skin lesions. Conversely, nearly 80% of the cases with fractures also had skin lesions. The location of the skin lesions frequently did not correspond to the fracture sites. This finding emphasizes the significance of hematomas and abrasions as early indicators for more severe injuries. In addition, the value of skeletal surveys in cases with suspicion for AHT is corroborated.

## Conclusions

The present study provides a confession-supported enlargement of the general database in the field of child physical abuse, especially as to fractures and skin lesions in AHT. The following conclusions can be drawn:

1. Our data strongly support the assumption that SBS/AHT is frequently accompanied by other forms of physical abuse, primarily blunt force trauma against trunk and extremities, since many fractures and skin lesions cannot be explained by AHT alone. Hence, the diagnosis of SBS (or AHT in general) should additionally be considered, if fractures and/or skin lesions are present in infants or toddlers.
2. The close relationship of fractures and skin lesions emphasizes once again the necessity of performing a complete skeletal survey when child physical abuse is suspected, and in particular when skin lesions of the head and trunk are present.
3. The present study illustrates the value of interdisciplinary collaboration in clinical forensic medicine, especially between forensic physicians, (pediatric) radiologists, and pediatricians.
